# A Novel Unsupervised Algorithm for Biological Process-based Analysis on Cancer

**DOI:** 10.1038/s41598-017-04961-6

**Published:** 2017-07-05

**Authors:** Tianci Song, Sha Cao, Sheng Tao, Sen Liang, Wei Du, Yanchun Liang

**Affiliations:** 1 College of Computer Science and Technology, Key Laboratory of Symbolic Computation and Knowledge Engineering of Ministry of Education, Jilin University, Changchun, 130012 China; 2 Computational Systems Biology Lab, Department of Biochemistry and Molecular Biology and Institute of Bioinformatics, University of Georgia, Athens, GA 30602 USA; 3 Zhuhai Laboratory of Key Laboratory of Symbolic Computation and Knowledge Engineering of Ministry of Education, Zhuhai College of Jilin University, Zhuhai, 519041 China

## Abstract

The aberrant alterations of biological functions are well known in tumorigenesis and cancer development. Hence, with advances in high-throughput sequencing technologies, capturing and quantifying the functional alterations in cancers based on expression profiles to explore cancer malignant process is highlighted as one of the important topics among cancer researches. In this article, we propose an algorithm for quantifying biological processes by using gene expression profiles over a sample population, which involves the idea of constructing principal curves to condense information of each biological process by a novel scoring scheme on an individualized manner. After applying our method on several large-scale breast cancer datasets in survival analysis, a subset of these biological processes extracted from corresponding survival model is then found to have significant associations with clinical outcomes. Further analyses of these biological processes enable the study of the interplays between biological processes and cancer phenotypes of interest, provide us valuable insights into cancer biology in biological process level and guide the precision treatment for cancer patients. And notably, prognosis predictions based on our method are consistently superior to the existing state of art methods with the same intention.

## Introduction

Many biological processes are rewired or reprogrammed during cancer initiation and progression. Therefore, identifying the involved biological processes and quantifying their levels of deregulation over a sample population are significant toward understanding cancer behaviors. Moreover, linking deregulation of biological processes in cancer patients with sensitivity to therapeutics that target the key components of these biological processes provides us an opportunity to guide personalized medicine^1, 2^. Advances in modern technology have enabled us to measure the mRNA abundance at a whole genome scale, and also these have led to comprehensive catalogs of diverse molecular functions underlying biological processes in cancers. Therefore, it is essential to design a method with capability of quantifying biological processes by integrating gene expression profiles to reveal the general aberrations residing in cancers, as well as enabling the patient specific deregulations of biological processes.

Nevertheless, there are at least two challenges. Firstly, in the era of high-throughput technology, we are constantly facing large-scale datasets enriched with information, but complex in the feature size and the intrinsic structure. And traditional methods using gene expression data in biological process analysis only target the single gene, such as oncogene or tumor-suppressor, while they cannot accurately and comprehensively unveil the variations as a result of combined effects of associated genes involved in a biological process. It is thus natural to transform gene level information into biological process level information so that we not only reduce the data dimensionality but also summarize the patterns of biological processes exhibited by the intrinsic structure of gene expression data themselves. Secondly, almost all the existing methods try to measure the activity of a biological process for entire sample^3–5^, but actually they do not provide any useful information on deregulation of that biological process in cancer. In order to guide personalized understanding of cancer biology, our method focuses on deducing independently, for every concerned biological process, a score that can represent the relative extent to which the pathway is deregulated in every individual sample.

For quantifying biological processes, there exist two popular frameworks. One of them characterizes the deregulation of biological process by deducing score for each sample based on network structure, such as PathOlogist^6^, as well as Pathway Recognition Algorithm using Data Integration on Genomic Models (PARADIGM)^7^. But the truth is that, at least for now, for some complicated biological process, we only have a rough understanding about its mechanism and relationship among its components, that is, we do not have a clear and complete map of the network structure. Moreover, lack of essential relevant data, such as protein data which PARADIGM requires, further limits usage of these methods in many occasions. Another framework does not rely on network information of biological process, such as Gene Set Variation Analysis (GSVA)^8^, which estimates variation of gene set belonging to a biological process over the samples. It starts evaluating differential expressed gene in each sample in the context of the current sample distribution, that is, constructing statistics of expression level and ranking these statistics for each sample on each gene, then condensing the statistics of gene expression level into biological process level scores by calculating sample-wise enrichment scores. However, GSVA is biased in scoring biological process where all genes have the same pattern, and yields less informative score for each sample^9^.

In this article, we have developed an unsupervised learning algorithm called Local Principal Curve (LPC), which is based on the theory of Principal Curve (PC)^10^, to quantify the variations of each biological process for each individual sample. Our algorithm successfully bypasses the shortages of the conventional approaches, such as explicitly encoding phenotypes within biological process scoring algorithms, or restricting to detailed knowledge of the network, and further concentrates on summarizing patterns in context-specific manner. Principal curve generalizes the principal component line, aiming at passing the middle of the data cloud, and further provides a smooth one-dimensional curved approximation to a set of data points in R^*p*^. The existing principal curve algorithms mainly take two following strategies: “top-down” strategy and “bottom up” strategy. The “top-down” strategy is usually used in traditional principal curve algorithm, it starts with a straight line, which is usually the first component of the whole data set, and tries to find the principal curve iteratively by minimizing the average squared distance between data points and corresponding projections to the curve. And the “bottom up” based strategy is applied in our LPC algorithm, it concatenates piece-wise curves that are determined by local neighborhood of currently considered data point. Comparing the two strategies, we find the second strategy seems to be more appropriate in finding principal curve, since the stretch of principal curve depends on the local distribution of data points rather than an initial line can effectively characterize complex data, such as circled and spiral data, even data with multiple branches. Our quantifying algorithm adopts the second strategy to construct principal curve characterizing the intrinsic structure of a biological process, through which we can obtain Biological Process Score (BPS) for each biological process and sample.

To demonstrate the biological process deregulation scores obtained using our method indeed capture biologically and clinically relevant information in a sensible manner, we applied these biological process scores to model survivals of breast cancer as a case study. With the BPSs yielded by our method as inputs, we constructed a biological process based model that selected several biological processes are highly correlated with clinical outcomes, and these BPSs perform best in prognosis prediction in comparison to existing methods. Moreover, we found that the biological relevancies with compelling evidences between selected or cancer hallmark related biological processes and breast cancer have been referred to in many existing researches.

## Results

### The Outline Overview

**Figure 1:**
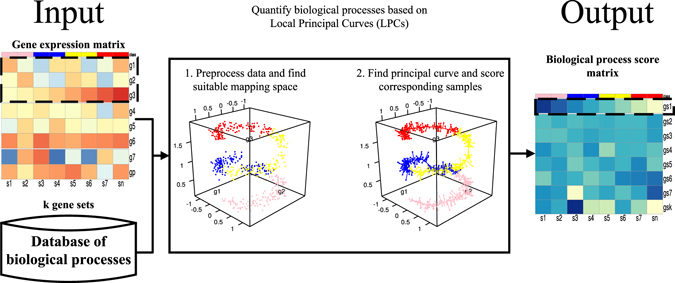
An example of quantifying biological processes based on Local Principal Curves (LPCs) algorithm. The input for the proposed method are a simulated gene expression matrix with *n* samples and *p* genes, and *k* biological processes we concern. 1) For selected biological process, we preprocess expression matrix of these three genes involved in by Principal Component Analysis (PCA), and then, and we then choose suitable Principal Components (PCs) to construct mapping space (see Method). To keep things simple, we here identify gene with PC. The plot intuitively illustrates the distribution of samples in mapping space consisting three PCs. 2) We stretch LPC with a starting point and compute projection indices for samples as Biological Process Scores (BPSs). (see Method). The plot illustrates a well-defined curve passes through data cloud with samples projecting onto corresponding position on the curve. The output of the proposed method is a matrix consisting of BPSs.

The outline of proposed algorithm is shown in Fig. 1. The input for biological process quantifying algorithm is a gene expression matrix with n samples and p genes, and k selected gene sets which are involved in specific biological processes. For each selected gene set, its corresponding expression matrix is preprocessed through Principal Component Analysis (PCA), to further maintain the stability and robustness of the following biological process scores. The suitable principal components are elaborately chosen and then used to construct mapping space (see Methods). With these principal components, each sample can be projected to mapping space as a data point. Then, the LPCs are stretched with a starting point that is usually the central point of reference samples, such as normal samples, or the point with the highest density among samples chosen through density estimation. They gradually proceed the data cloud in a localized way until reaching the data cloud’s boundary (see Methods). And further, we use the length along the LPC between the endpoint and the point of associated sample after projecting onto the LPC to estimate BPS (see Methods). The output of our method is matrix consisting of BPSs.

### Model based on BPS matrix performs best in identifying biological processes predictive of cancer survival

In this article, we first applied our method on a breast cancer dataset GSE3494 with abundant clinical information that includes 236 samples over 1330 biological processes, and obtained corresponding BPS matrices (see Method). To validate the effectiveness and robustness of our BPS matrix, we compared our BPS matrix with PAM50 gene expression matrix^11^ and pathway-based deregulation score (PDS) matrix yielded by Pathifier^12^ respectively. Here, PAM50 is a gene set designed for subtyping breast cancer, and also has satisfactory performance in breast cancer prognosis^13^, and Pathifier is derived with the same intention as ours, but using principal curve method with classical “top-down” strategy, and was believed to be the best currently available method for deducing gene set level scores^14^.

**Figure 2:**
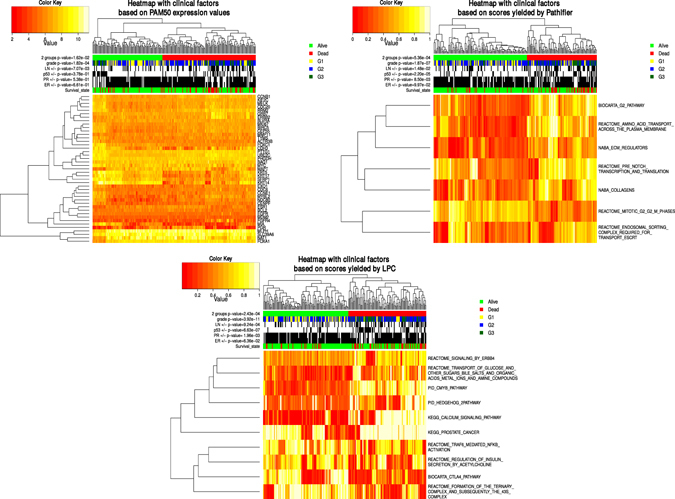
The comparison of the association between selected biological processes and clinical outcomes among gene-based model and biological process-based models. The samples are divided into two groups by performing the hierarchical clustering on the PAM50 gene expression matrix, PDS matrix yielded by Pathifier and BPS matrix yielded by LPC of selected biological processes, respectively. Green and red colors represent alive and dead survival status of samples respectively, White and black colors are associated with positive and negative status of ER, PR, p53 mutation, and lymph node of samples respectively. Yellow, blue and dark green colors denote different grades of samples respectively. The p-values of these clinical outcomes and dichotomized two groups with relation to survival status are calculated using Chi-square tests.

We selected those biological processes whose scores are significantly associated with breast cancer survival. To do so, we applied L1-LASSO penalized multivariate Cox proportional hazards (Cox-PH) model^15^ on the BPS matrix, PAM50 gene expression matrix and PDS matrix respectively with sample survival status as the response variable, and scores of each biological process or expression values of each gene as predictor variables. And then we calculated the significances of associations between the three matrices and various clinical outcomes. The scores for the three matrices and their associations with various clinical outcomes are shown as heatmaps in Fig. 2.

Compare to PAM50 gene wise expression matrix and PDS matrix by the same survival analysis model respectively, BPS matrix is more prognostic for survival status, meanwhile, has high correlation with clinical outcomes. This conclusion can be confirmed by following two observations: 1) Dichotomized samples of high risk and low risk group through hierarchical clustering of BPS matrix have higher correlation to survival status (Chi-square test p-value = 2.43e-4), in comparison with PAM50 gene expression matrix (Chi-square test p-value = 1.62e-2) and PDS matrix (Chi-square test p-value = 5.36e-4). 2) Clinical factors such as ER status, PR status, p53 mutation status, lymph node status and grade, have stronger association with those two groups dichotomized by clustering BPS matrix (Chi-square test p-value 6.36e-2, 1.96e-3, 6.63e-7, 9.24e-4 and 3.92e-11), in comparison to PDS matrix (Chi-square test p-value 9.97e-2,8.50e-3, 2.20e-5, 1.48e-2 and 1.87e-7) and PAM50 matrix (Chi-square test p-value 6.61e-1, 5.38e-1, 3.78e-1, 7.07e-3, 1.82e-4).

To further validate the effectiveness and robustness of BPS matrix, we introduce Prognostic Index (PI) value, which is logarithm of hazard ratio obtained from L1-LASSO COX-PH model, as a measure of survival risk for each sample (see Methods). Sample with higher PI value tends to have a severe survival status. Therefore, we evaluated the effectiveness and robustness of BPS matrix via the following two approaches involving in PI: 1) Estimating survival difference between low risk and high risk groups dichotomized by PI threshold, via calculating Wilcoxon log rank test p-value of Kaplan-Meier survival curves based on these two groups. 2) Calculating area under the curve (AUC) of ROC based on binary classification through treating PI as a likelihood of survival status.

**Figure 3:**
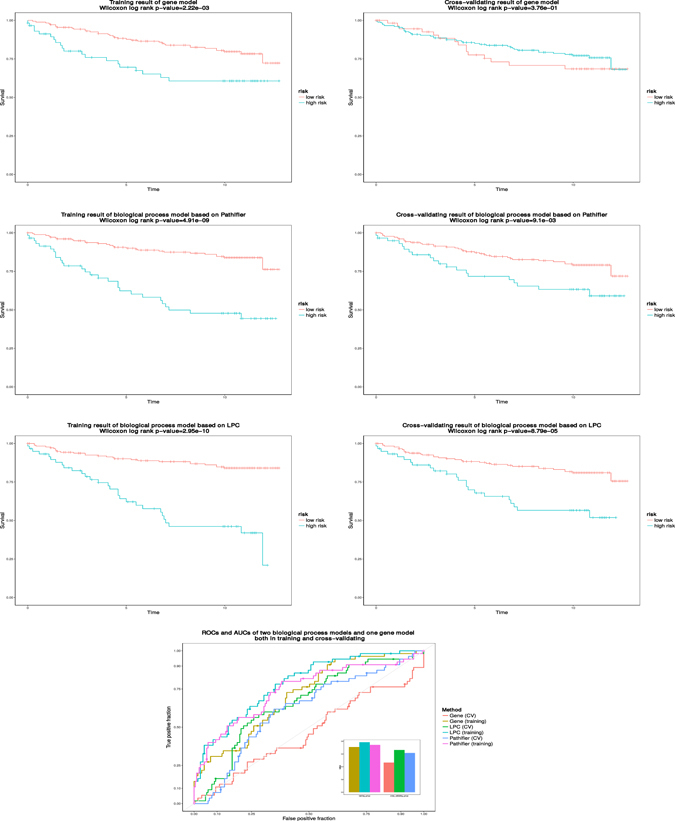
The comparison of the prognosis performance among gene-based model and biological process-based models on GSE3494 dataset. The prognosis indices (PIs) for all samples in the dataset are calculated with associated models, and applied to dichotomize the samples into high and low risk groups in comparison to PI cutoff. The p-values of the survival difference between the two groups are calculated using Wilcoxon log-rank tests, and (+) denotes the censored observations. The ROC curves are generated by regarding PI values as predictions in comparison to survival status of samples. Leave one out cross validation (LOOCV) are performed to calculate Wilcoxon log rank p-values and AUCs across models. We found the Wilcoxon log rank p-values and the AUCs derived from the model based on BPS matrix yielded by LPC outperform than those derived from the models based on PAM50 gene expression matrix and PDS matrix yielded by Pathifier both in training and cross validating results.

We applied leave one out cross validation (LOOCV) to assess the effectiveness and robustness of BPS matrix. As to the first approach, we trained L1-LASSO COX-PH model using training samples at each fold, and then predicted PI value for test sample using the coefficients fitted in the training model. Test sample was further labeled with either low or high risk through comparing its predicted PI to PI threshold, which was determined from the training model to match the ratio of alive versus dead samples of training and used to dichotomize the training samples into higher risk and lower risk groups. Then, we calculated Wilcoxon log rank test p-values of Kaplan-Meier survival curves based on these two risk groups for training result (2.95e-10) as well as LOOCV result (8.79e-5) respectively. By contrast, we obtained the Wilcoxon log rank test p-values from PAM50 gene expression matrix for training result (2.22e-3) as well as LOOCV result (3.76e-1), and from PDS matrix for training result (4.91e-9) as well as LOOCV result (9.1e-3) using the same procedure described above respectively. As to the second approach, we regarded PI as likelihood to survival status, and calculated AUCs for training result (0.78) as well as LOOCV result (0.69). In contrast, we also calculated AUCs for training result (0.71) as well as LOOCV result (0.47) based on PAM50 gene expression matrix, and for training result (0.74) as well as LOOCV result (0.62) based on PDS matrix, see Fig. 3. The BPS matrix shows the best results both in significance of correlation with clinical factors and performance of prognosis prediction. And we conducted the same experiments on other two datasets, GSE1456 and GSE4922, BPS matrix consistently performs better than PAM50 gene expression matrix and PDS matrix. (see Supplementary Figures S1 and S2).

Moreover, we further evaluated the effectiveness and robustness of BPS matrix considering batch effect across datasets. We used one dataset (GSE3494) as training data, and two other independent datasets (GSE1456 and GSE4922) as testing data. As to the first approach, we trained L1-LASSO COX-PH model using the training data, and then predicted PI value for test data using the coefficients fitted in training model. All samples in the training data were divided by PI threshold into low risk and high risk groups, where the ratio of these two risk groups matches the ratio of alive versus dead samples in training data. And the same PI threshold was applied to dichotomize the testing data. We repeated the experiments based on the above two approaches and examined the results from two independent datasets, then found that the Wilcoxon log rank test p-values of Kaplan-Meier survival curves from BPS matrices of testing data (4.42e-2 and 4.79e-2) are more significant in comparison to those from PAM50 gene expression matrices (1.5e-1 and 3.26e-1) and PDS matrices (5.3e-1 and 7.65e-1), and the AUCs from BPS matrices of testing data (0.54 and 0.57) are better than those from PAM50 gene expression matrices (0.42 and 0.53) and PDS matrices (0.40 and 0.55). The BPS matrices still consistently show the best results both in significance of correlation with clinical factors and performance of prognosis prediction (see Supplementary Figure S3).

### The relevance of selected biological processes to cancer

As for biological processes selected, we further explored their associations to related cancer. In this article, we used breast cancer as a case study and found that some of biological processes related to signal transduction are observably highlighted. One of these signaling biological processes is REACTOME SIGNALING BY ERBB4, where ERBB4, also known as HER4, belongs to epidermal growth factor receptor (EGFR) subfamily of receptor tyrosine kinases. Supporting role in promoting growth of breast cancer cells, signaling by ERBB4 also has been suggested to induce differentiation and apoptosis of breast cancer cells^16, 17^. The second one is KEGG CALCIUM SIGNALING PATHWAY, which plays an important role in the regulation of a variety of key processes in breast tumorigenesis such as proliferation, migration, invasion, cell death and angiogenesis^18, 19^. Also, it is worthy to note that the alterations of calcium signaling pathway are widely observed among different breast cancer subtypes, which are mediated by different mechanisms and result in different functionality changes^20, 21^. And the third one is PID HEDGEHOG 2PATHWAY, which transmits information such as time and position dependent expression patterns to embryonic cells required for proper development. It plays a crucial role in vertebrate embryogenesis by controlling cell fate, patterning, proliferation, survival and differentiation, and thus affects development from embryonic stage^22, 23^. Aberrant activation of the hedgehog signaling pathway has clearly been tied to breast cancer development and progression^24, 25^. The last but not the least one is KEGG P53 SIGNALING PATHWAY. Its functionality in breast cancer has been extensively reviewed regarding to its activation in response to cellular stress, initiating DNA repair and cell cycle arrest, as well as regulating apoptosis, metastasis and angiogenesis^26, 27^.

Then, we focused on biological processes about genetic information processing. The first one is PID CMYB PATHWAY, where c-MYB is the prototype member of a small family of transcription factors involved in cell survival, proliferation, differentiation and transformation, and it has been reported recently that c-MYB enhances the migration and invasion in breast cancer^28^. The second one is REACTOME TRAF6 MEDIATED NFKB ACTIVATION, where NF-kB is a family of transcription factors that play critical roles in cell survival, proliferation, inflammation and immunity^29^. And, there are many evidences show the activation of NF-kB contributes to the breast cancer development and progression^30^.

And the rest of biological processes still remain strong associations to breast cancer. One of them should be noticed is BIOCARTA CTLA4 PATHWAY, where Cytotoxic T lymphocyte antigen-4 (CTLA-4) is a key negative regulator of T cell activation. Within hostile local microenvironment and in distant organs, immune cells such as T cell and their mediators are known to facilitate metastasis formation^31, 32^. It has been demonstrated that the blockade of CTLA-4 augments endogenous responses to tumor cells, thus leading to tumor cell death in breast cancer^33^. And another one needs to be considered is REACTOME REGUALTION OF INSULIN SECRETION BY ACETYLCHOLINE, insulin of high level induces proliferative tissue abnormalities because of its strong anabolic effect of insulin, which results in stimulated DNA synthesis and cell proliferation^34^.

### The relevance of selected cancer hallmark related biological processes to cancer

In this article, we also applied our method on the same breast cancer dataset over 229 cancer hallmark related biological processes (see Methods), and then obtained corresponding BPS matrix. And we then did the same experiment on this BPS matrix of cancer hallmark related biological processes, even though the correlation with clinical outcomes and prognosis prediction are slightly inferior to those derived from BPS matrix of biological processes from MsigDB above, it still performs better in comparison to either PAM50 gene expression matrix or PDS matrix of cancer hallmark related biological processes (see Supplementary Figures S4 and S5).

In the following part, we checked 6 biological processes from above survival model that are highly correlated to the malignancy (progression, invasion and metastasis) of breast cancer. Firstly, the genome instability is one of the most fundamental hallmarks of cancer, while the base excision repair pathway is the “gatekeeper” biological process to ensure the genome integrity. It is widely observed that the deficiency of this biological process associates with the malignancy of breast cancer patients^35^. Furthermore, epithelial to mesenchymal transition (EMT) confers the trait of advanced or late stage cancer, which is induced by the redirection of TGF-β signaling^1^. The EMT program in breast cancer is also reported to be highly associated with cancer invasion and metastasis^36^, hence it is valuable to evaluate the related biological processes in our analysis. We then analyzed the N-acetly gulcosaminyl transferase pathway, whose synergistic effect with TGF-β to induce EMT in breast cancer is reported^37^. Moreover, SMAD and its downstream target signaling pathway is also highlighted, where SMAD acts as the intracellular effector of TGF-β signaling pathway^38^. In addition, the extracellular matrix cell to cell adhesion pathway (EGS_Extracellular_Matrix_Cell_Adhesion) and the global intercellular signaling pathway (EGS_Signaling_Pathways) are included, both of which are reported to be highly associated with the invasion and metastasis of breast cancer^39^. At last, we also checked the immune detection pathways, especially the humoral immune responses, since this biological process is widely reported to have the anti-tumor role and its deficiency is observed during the breast cancer progression^40^.

## Discussion

In this article, we propose a novel unsupervised algorithm based on local principal curve in acquiring intrinsic structure of biological process and quantifying corresponding biological process for each sample, which successfully bypasses the conventional method of explicitly modeling phenotypes of samples and network information of biological process within algorithm itself. The obtained biological process scores provide superior performance in survival analysis compared to scores yielded by Pathifier and expression values of PAM50 genes. Moreover, the selected biological processes in survival analysis are highly associated with breast cancer and even cancer hallmarks, and further can be exploited to target prognosis of breast cancer and even gain an insight into progress and development of breast cancer.

Notably, our algorithm is highly potential in detecting the complicated structure of biological process, including spiral, bifurcation and even multiple disconnected branches. But in this article, we are constrained by gene expression data with insufficient samples, that is, there exist large gaps among different types of samples in some mapping spaces of biological processes. We will try to collect more samples in further study to more accurately and completely capture the subtle changes. And even if samples are adequate, they may be unevenly distributed in mapping space of a specific biological process, we cannot reach a comprise between characterizing subtle information and picturing the whole underlying structure of a biological process when using the same step and bandwidth in each movement of localized center of mass. Therefore, we will further work on improving our algorithm in selecting a data-adaptive parameter dynamically. As for values of the deregulation scores produced by our method, they can only be acted as an estimation of the relative extent to which the behavior of biological process in a sample deviates from the designated sample, we still cannot use these scores to mine the real changes, such as up- and down- regulation, or fluctuations over time in biological process. We will further make efforts to find a reasonable way to address this issue.

Furthermore, there still exists some information cannot be reflected in individual omics data, such as gene expression data that we used in this article. It is necessary to consider extending our algorithm to integrate multiple omics data in a reasonable manner. More efforts have been made in modeling cancer hallmarks with their network for gain some insights into cancer biology, and they have generated many interesting results^41–43^. Next, we will try to link our scores of the cancer hallmark related biological processes to their networks for each individual patient, and generally infer the process of the changes over variation of scores in these cancer hallmark related network, which will have substantial impact on understanding the mechanisms of tumorigenesis and cancer development, even guiding personalized treatment and prevention of cancer.

## Methods

### Data Summary

In this study, we collected three breast cancer datasets GSE3494^44^, GSE1456^45^, GSE4922^46^ from public database Gene Expression Omnibus (GEO)^47^. All datasets are measured on microarray platform of Affymetrix HG-U133A, including 236, 159, 249 samples with survival information respectively, and GSE3494 has more abundant clinical outcomes, such as status of Estrogen Receptor (ER), Progesterone Receptor (PR), Lymph node, P53 mutation and Grade etc. The annotated gene sets are obtained from Molecular Signatures Database (MSigDB)^48^. We used curated gene sets collection (C2 collection), which includes 217 biological processes from BIOCARTA^49^, 186 biological processes from KEGG^50^, 674 biological processes from REACTOME^51^ and 253 biological processes from Pathway Interaction Database^52^. And all cancer hallmark related gene sets we used in this article are gathered from literatures and databases by ourselves (see Supplementary Data files).

### Data preprocessing and finding suitable mapping space

Firstly, the variation of expression values may be largely affected by gene wide range scales. Hence, genes of absolute smaller variations caused by its intrinsic lower expression scales, are usually overwhelmed by those genes of higher expression scales. To eliminate this bias induced by different scales of gene expression values, we normalize gene expression values of tumor samples by first subtracting each gene’s expression value of each tumor sample by the mean expression value of the normal samples for the corresponding gene, and then dividing each gene’s expression value of each tumor sample by the variance of the normal samples for the corresponding gene when normal samples exist, otherwise, we normalize gene expression values of tumor samples by calculating z-scores. Here, to reduce technological noise or other biases, the gene expression data are processed using limma^53^ before our normalization procedure. And we reduce the batch effect across the datasets using Combat^54^. Each of normalized gene expression values is regarded as a degree to which a tumor sample deviates away from its corresponding normal samples in current gene expression scale. Besides, to avoid genes whose variations are attributable to noise, we exclude the genes whose variances lower than 25% of all genes.

In addition, it is noteworthy that some genes in the same biological process may be highly correlated, resulting in the redundant information that causes higher computation expense. However, some important information regarding to the functionalities of the biological processes may hidden in these genes. To counter this issue, we decide not to search principal curve for each biological process in its corresponding original space *S* ^*p*^ denoted by all genes involved in that biological process, but in the subspace S′_*p*_ of *S*_*p*_ spanned by first *k* principal components from gene expression data of that biological process. Here, *k* is identified by the number of principal components along which the variances exceed by more than 10% of those in normal samples.

### Finding local principal curves and calculating projection index

Assume that we have a data set *X*_1_, …, *X*_*n*_, with *X*_*i*_ ∈ R^*p*^. Local principal curves (LPCs)^38^ are based on the idea that, at each point *x* ∈ R^*p*^ along a principal curve, the localized first principal component line forms approximately a tangent to the curve. Beginning with a starting point, *x* = *x*_0_ ∈ R^*p*^, LPCs go successively through the data cloud, alternating between the following three steps:

1. Calculating a localized center of mass ${\mu }^{x}={\sum }_{i=1}^{n}{w}_{i}{X}_{i}$, where ${w}_{i}={K}_{H}({X}_{i}-x){X}_{i}/{\sum }_{i=1}^{n}{K}_{H}({X}_{i}-x);$
2. Computing the first local eigenvector *γ* ^*x*^ of local weighted covariance matrix of *x*, $\mathop{\Sigma }\limits^{x}={({\sigma }_{jk}^{x})}_{(1\le jk\le p)}$, where ${\sigma }_{jk}^{x}={\sum }_{i=1}^{n}{w}_{i}({X}_{ij}-{\mu }_{j}^{x})({X}_{ik}-{\mu }_{k}^{x})$ and ${\mu }_{j}^{x}$ denotes the *j*-th component of *μ* ^*x*^;
3. Using a predetermined step size *t*_0_ to iterate from *μ* ^*x*^ to *x* = *μ* ^*x*^ + *t*_0_ *γ* ^*x*^ along the direction of *γ* ^*x*^.
4. Stop forwarding when the boundary of data cloud is reached, or the difference of the previous and the current center of mass *μ* ^*x*^ falls below a preliminary threshold.

The sequence of local centers of mass *μ* ^*x*^ forms the local principal curve. Here, ${K}_{H}(\cdot )={|H|}^{-1/2}K({H}^{-1/2}\cdot )$, with a multivariate kernel *K* and a positive definite bandwidth matrix $H={\rm{diag}}({h}_{1}^{2},\ldots ,{h}_{p}^{2})$, which steers the size of local neighborhood and exhibits a strong influence on resulting estimate local centers of mass. The value of bandwidth matrix can be selected through coverage properties of locating local centers of mass discussed in Einbeck *et al*.^55^. In this article, we arbitrarily choose 10% of range in each dimension for bandwidth matrix. Extensions to disconnected and branched curves are also considered in Einbeck *et al*.^56^, and easily implemented by using suitable multiple starting points. The issue of crossing and oversteering can be handled by using angle penalization^57^. As only points in the local neighborhood are considered in each iteration, the algorithm is quite flexible, and robust to noisy data patterns.

For a fitted LPC consisting of *L* local centers of mass ${\mu }^{{x}_{\ell }}={({\mu }_{1}^{{x}_{\ell }},\ldots ,{\mu }_{p}^{{x}_{\ell }})}^{T}$, $\ell =1,\ldots ,\,L$, we try to seek a curve $\{g(t),t\in {I}_{g}\}$ which interpolates the local centers of mass. And this curve can be parameterized by the function below:

1 $${I}_{g}\to {{\rm{R}}}^{p},t\mapsto {({g}_{1}(t),\ldots ,{g}_{p}(t))}^{T}$$

where *I*_*g*_ ∈ R denotes the domain of *g*, and *g* is a vector function with *p* coordinates, each smooth function shares the same parameter *t*. The parameter *t* corresponds to the projection index, whose variation traces all points along the curve. One ending point of the curve is chosen to be the origin corresponding to *t* = 0.

1. Computing a discrete, preliminary parameterization $\,{({s}_{\ell })}_{(1\le \ell \le L)}$, with the same origin as *t*, by adding up the Euclidean distances between subsequent ${\mu }^{\ell },\ell =1,\ldots ,L$;
2. For each dimension *j* = 1, …, *p*, interpolating the points ${({s}_{\ell },{\mu }_{\ell }^{j})}_{(1\le \ell \le L)}$ by a cubic spline, yielding graphs $(s,{\mu }_{j}(s))$. Integrating them together to obtain a continuous and differentiable spline function ${({\mu }_{1},\ldots ,{\mu }_{p})}^{T}(s)\equiv \mu (s)$.
3. For each value of *s* within the support of the spline function, recalculating the parameter using the arc length,

2 $$t={\int }_{0}^{s}\sqrt{{({\mu }_{1}(u))}^{2}+\ldots +{({\mu }_{1}(u))}^{2}}du$$

and setting *g*(*t*) = *μ*(*s*). It should be noted that smoothing is not involved in previous steps. Once this parameterization is established, each data point *x*_*i*_, *i* = , …, *n* can be projected onto the curve through finding the point on the curve which is nearest to it in Euclidean distance, meanwhile, yielding the projection index *t*_*i*_.

### Scoring biological process and maintaining stability of biological process scores

Now, we suppose that there exists a *p*-dimensional space ${S}_{p}^{^{\prime} }$, where each point in this space represents a sample with corresponding component scores obtained from gene expression data after performing PCA. In accordance with our method, we indeed get a one-dimensional curve passing through the “middle of data cloud”, which best summarizes the intrinsic structure of our data and quantifies all samples through projecting these samples onto curve itself. And subsequently, we compute their corresponding projection indexes, which further characterize the variability of the samples across ${S}_{p}^{^{\prime} }$ and provide us the chance to find the hidden factors (e.g. tumor progression) that comes from the variance of the biological process. Here, we assume that any two samples in ${S}_{p}^{^{\prime} }$ that have proximal projection onto the curve have similar biological functions.

As to the scoring biological process, we perform LPC based on “bottom-up” strategy mentioned above to find a principal curve in ${S}_{p}^{^{\prime} }$ with as starting point *x*_0_ (In fact, when there is no designated starting point, we will choose the point of highest density by applying mean shift on all points). Once such a principal curve is found, we project each data point *x*_*i*_ representing *i*-th sample onto *f*_*i*_ on the curve, and then calculate projection index *t*_*i*_ along the curve between *f*_*i*_ and the projection of one end point of curve *f*_0_ (as a matter of fact, we set *f*_0_ as *x*_0_ when there are normal samples as references). Then, we normalize all projection indices as biological process scores *BS*_*i*_, each of which measures the degree to which a given biological process of sample *i* is altered by tumor (see Fig. 1).

Similarly, some genes residing in a same biological process convey less useful information but keep high variations in seeking principal curves. For example, due to individual differences of samples or noises, their variations do not reflect information relevant to biology we are trying to capture. The principal curves may overwhelm subtle changes in biological process of tumors and disturb to keep the stability of principal curves. With respect to this situation, we prefer omitting these genes to avoid possible adverse outcomes above. Actually, principal components are linear combinations of genes, and the same considerations imply that we are also able to omit some redundant principal components from first *k* principal components to filter those noisy genes and evade finding unstable principal curves.

### Survival analysis

In this article, we use the COX-PH model for survival analysis with the assumption that log hazard ratios are constant over time for features. Suppose that we have a matrix **X**_*p*×*n*_ with each row representing a feature and each column representing a sample, COX-PH model characterizes the relationship between survival status and **X**_*p*×*n*_ as following:

3 $$h(t|{\bf{X}})={h}_{0}(t){e}^{{\beta }^{T}{\bf{X}}}$$

where *h*_0_(*t*) is a hazard function only depends on time as baseline, *β* is a coefficient vector of corresponding features. Subsequently, the relative hazard ratio between any two features **X**_*i*_, **X**_*j*_ (1 ≤ *i*, *j* ≤ *p*) is independent on time and only determined by the differences of two features:

4 $$\frac{h(t|{{\bf{X}}}_{i})}{h(t|{{\bf{X}}}_{j})}={e}^{{\beta }_{i}{{\bf{X}}}_{i}-{\beta }_{j}{{\bf{X}}}_{j}}$$

We finally decide to use PI as an indicator of survival risk for individual sample to further evaluate the effectiveness and robustness of BPSs in survival analysis, since for any sample **X** ^*k*^ (1 ≤ *k* ≤ *n*) at one time, PI is relative constant to other samples, and can be represented as following:

5 $$P{I}^{k}={\beta }^{T}{{\bf{X}}}^{k}$$

## Electronic supplementary material

Supplementary Information

Dataset 1
