# Supplementary material for: A Novel Unsupervised Algorithm for Biological Process-based Analysis on Cancer
Source: Sci Rep. 2017 Jul 5;7:4671. doi: 10.1038/s41598-017-04961-6 (PMC5498659; doi:10.1038/s41598-017-04961-6)
Supplement: Supplementary file 1 — Supplementary Information [file 41598_2017_4961_MOESM1_ESM.pdf]

A Novel Unsupervised Algorithm for Biological Process-based Analysis on Cancer

Tianci Song[1], Sha Cao[2], Sheng Tao[2], Sen Liang[1, 2], Wei Du[1, 2 *], Yanchun Liang[1, 3 *]

[1] College of Computer Science and Technology, Key Laboratory of Symbolic Computation and Knowledge Engineering of Ministry of Education, Jilin University,

Changchun, 130012, China

[2] Computational Systems Biology Lab, Department of Biochemistry and Molecular Biology and Institute of Bioinformatics, University of Georgia,

Athens, GA 30602, USA

[3] Zhuhai Laboratory of Key Laboratory of Symbolic Computation and Knowledge Engineering of Ministry of Education, Zhuhai College of Jilin University,

Zhuhai, 519041, China

*** Corresponding authors: weidu@jlu.edu.cn; ycliang@jlu.edu.cn**

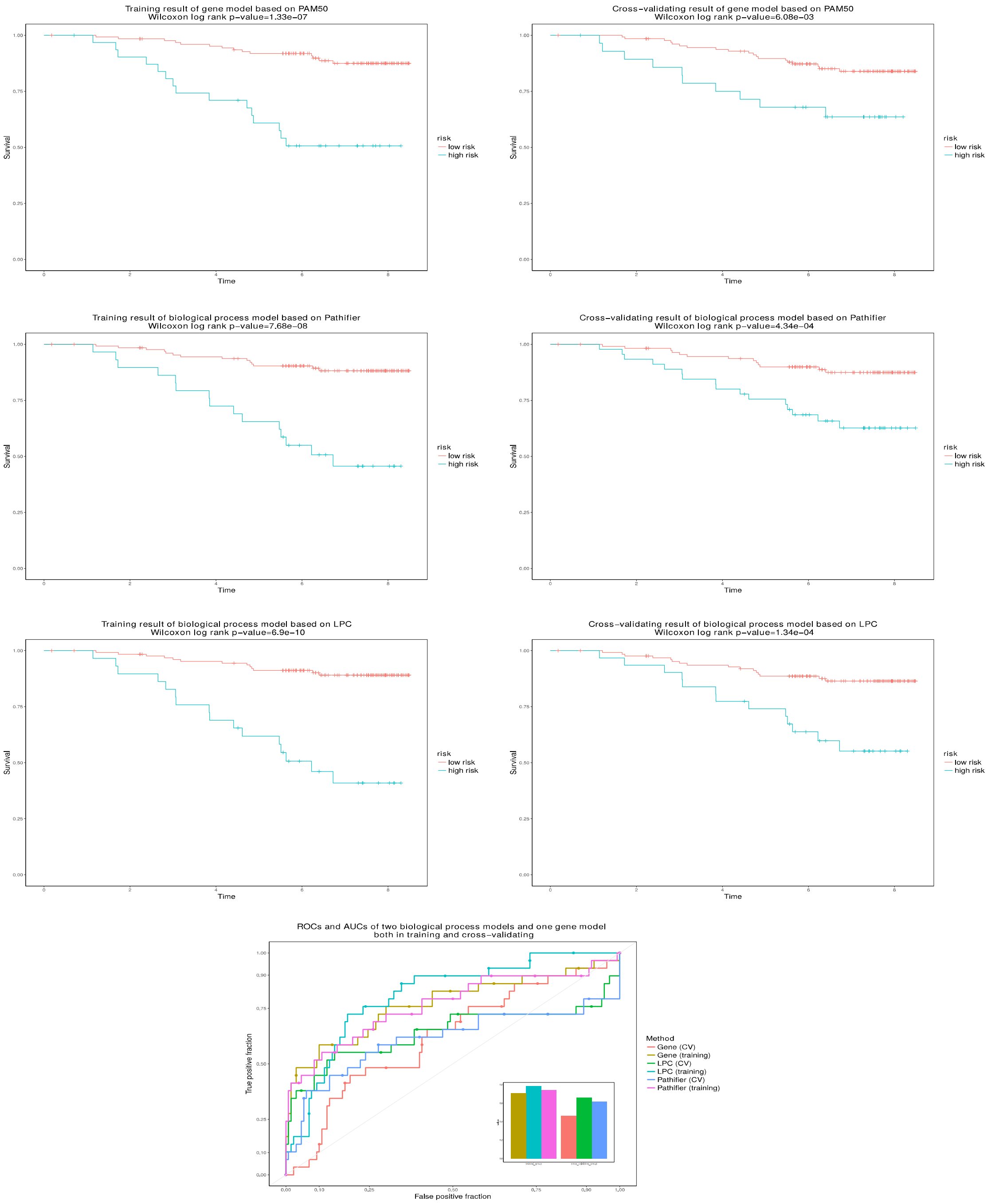

Figure S1. **The comparison of the prognosis performance among gene-based model and biological process-based models on GSE1456 dataset.** The prognosis indices (PIs) for all samples in the dataset are calculated with associated models, and applied to dichotomize the samples into high and low risk groups in comparison to PI cutoff. The p-values of the survival difference between the two groups are calculated using Wilcoxon log-rank tests, and (+) denotes the censored observations. The ROC curves are generated by regarding PI values as predictions in comparison to survival status of samples. Leave one out cross validation (LOOCV) are performed to calculate Wilcoxon log rank p-values and AUCs across models. We found the Wilcoxon log rank p-values and the AUCs derived from the model based on BPS matrix yielded by LPC outperform than those derived from the models based on PAM50 gene expression matrix and PDS matrix yielded by Pathifier both in training and cross validating results.

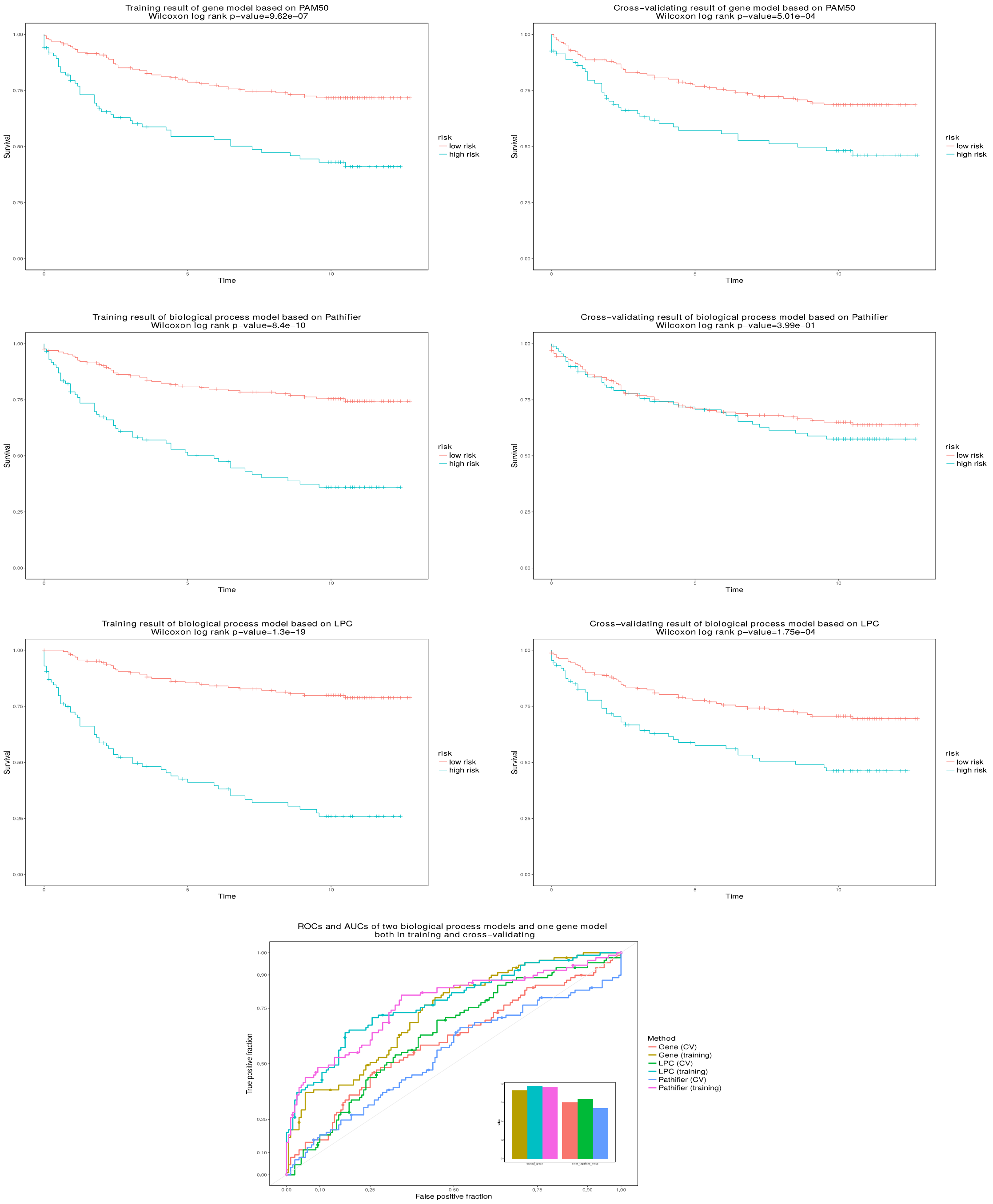

Figure S2. **The comparison of the prognosis performance among gene-based model and biological process-based models on GSE4922 dataset.** Similar to Figure S1. We found the Wilcoxon log rank p-values and the AUCs derived from the model based on BPS matrix yielded by LPC outperform than those derived from the models based on PAM50 gene expression matrix and PDS matrix yielded by Pathifier both in training and cross validating results.

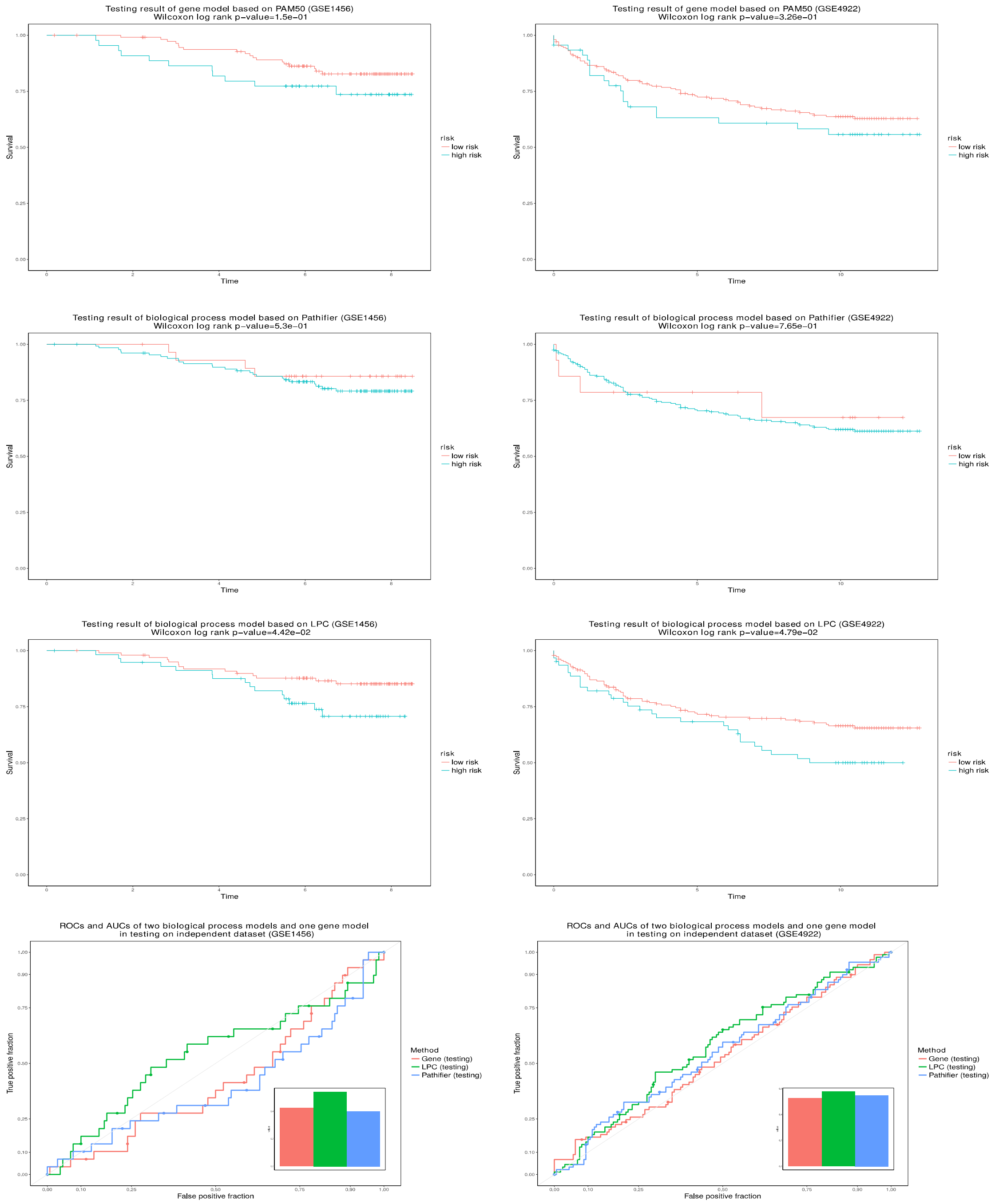

Figure S3. **The comparison of the prognosis performance among gene-based model and biological process-based models on two independent datasets.** Similar to Figure S1. We trained model using the dataset GSE3494 and tested on the two independent datasets GSE1456 and GSE4922. We found the Wilcoxon log rank p-values and the AUCs derived from the model based on BPS matrix yielded by LPC outperform than those derived from the models based on PAM50 gene expression matrix and PDS matrix yielded by Pathifier in testing results of two independent datasets.

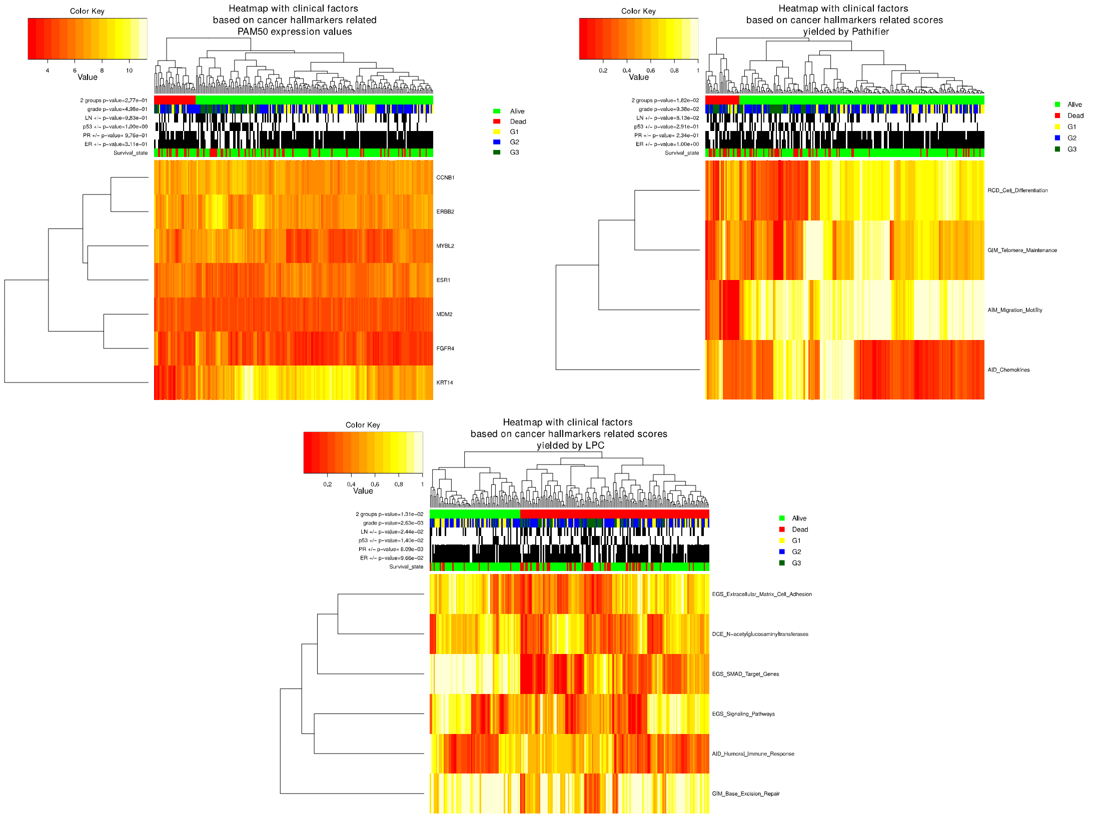

Figure S4. **The comparison of the association between selected biological processes of cancer hallmarks and clinical outcomes among gene-based model and biological process-based models**. The samples are divided into two groups by performing the hierarchical clustering on the PAM50 gene expression matrix, PDS matrix yielded by Pathifier and BPS matrix yielded by LPC of selected biological processes of cancer hallmarks, respectively. Green and red colors represent alive and dead survival status of samples respectively, White and black colors are associated with positive and negative status of ER, PR, p53 mutation, and lymph node of samples respectively. Yellow, blue and dark green colors denote different grades of samples respectively. The p-values of these clinical outcomes and dichotomized two groups with relation to survival status are calculated using Chi-square tests.

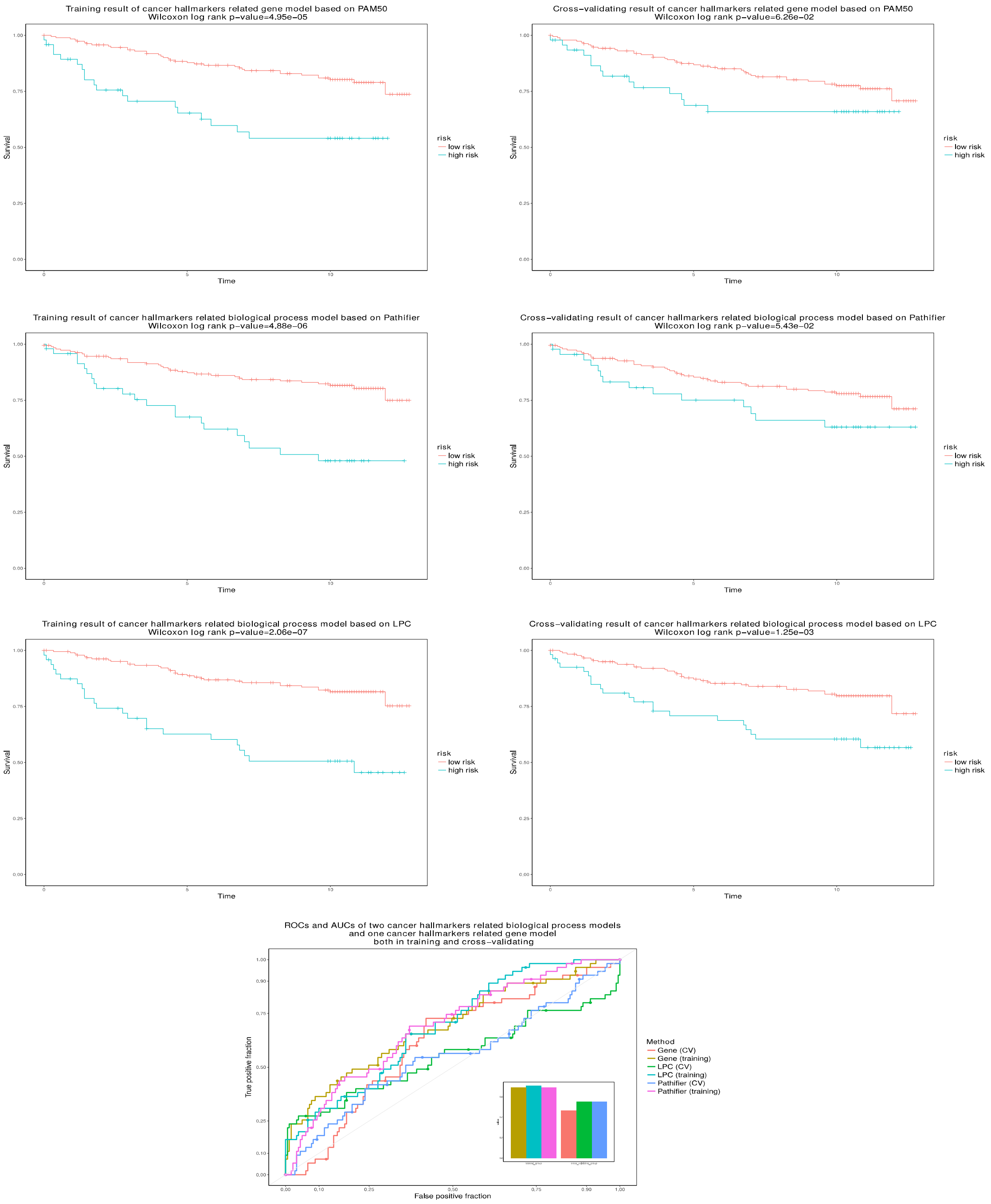

Figure S5. **The comparison of the prognosis performance among gene-based model and biological process-based models on GSE3494 dataset over cancer hallmark related biological processes.** Similar to Figure S1. We found the Wilcoxon log rank p-values and the AUCs derived from the model based on BPS matrix yielded by LPC outperform than those derived from the models based on PAM50 gene expression matrix and PDS matrix yielded by Pathifier both in training and cross validating results.
